# Rice Transcriptome Analysis Reveals Nitrogen Starvation Modulates Differential Alternative Splicing and Transcript Usage in Various Metabolism-Related Genes

**DOI:** 10.3390/life11040285

**Published:** 2021-03-27

**Authors:** Saurabh Chaudhary, Meenu Kalkal

**Affiliations:** 1 Cardiff School of Biosciences, Cardiff University, Cardiff CF10 3AX, UK; 2 Parasite-Host Biology, National Institute of Malaria Research, Dwarka, New Delhi 110077, India; meenu@mrcindia.org

**Keywords:** alternative splicing, gene expression, N-starvation, NUE, metabolism, transcript usage, rice

## Abstract

Nitrogen (N) is crucial for plant growth and development; however, excessive use of N fertilizers cause many problems including environmental damage, degradation of soil fertility, and high cost to the farmers. Therefore, immediate implementation is required to develop N efficient crop varieties. Rice being low nitrogen use efficiency (NUE) and a high demand staple food across the world has become a favorite crop to study the NUE trait. In the current study, we used the publicly available transcriptome data generated from the root and shoot tissues of two rice genotypes IR-64 and Nagina-22 (N-22) under optimum N supply (N^+^) and chronic N-starvation (N^−^). A stringent pipeline was applied to detect differentially expressed genes (DEGs), alternatively spliced (DAS) genes, differentially expressed transcripts (DETs) and differential transcript usage (DTU) transcripts in both the varieties and tissues under N^+^ and N^−^ conditions. The DAS genes and DTU transcripts identified in the study were found to be involved in several metabolic and biosynthesis processes. We suggest alternative splicing (AS) plays an important role in fine-tuning the regulation of metabolic pathways related genes in genotype, tissue, and condition-dependent manner. The current study will help in understanding the transcriptional dynamics of NUE traits in the future.

## 1. Introduction

Nitrogen (N) is a constituent of many biomolecules including amino acids, enzymes, growth hormones, nucleic acids, and proteins; its uptake from the soil, transportation among tissues, and metabolism is crucial to understand in plants. Nitrate (NO_3_^−^) and ammonium (NH_4_^+^) ions are the two most important forms of inorganic N required for plant growth and development [1]. NO_3_^−^ is considered as the major source of N for land plants while NH_4_^+^ is important for plants growing under anaerobic conditions like rice [2,3]. In agricultural practices, urea is the major N fertilizer that rapidly degrades into NH_4_^+^ and carbon dioxide (CO_2_) by enzyme ureases present in the soil [4]. Since N fertilizers have become a major source for the improvement of crop yields, their application to the agriculture fields has increased drastically in the recent past. It is estimated that out of the total N applied, only 30–40% is used or taken up by crops [5,6], whereas the remaining unused N is responsible for damaging the environment along with having an adverse effect on soil fertility [7,8]. Additionally, the ever-increasing world population requires more food; to meet up the demands an increase in agriculture productivity with minimal use of N fertilizers is required. Therefore, the current scenario demands immediate implementation of developing crops with high nitrogen use efficiency (NUE). The increased NUE in crops not only minimizes the utilization of N fertilizers but also increases the yield in the limited supply of N. In the past few years, various efforts have been made to develop crop varieties with high NUE using classical breeding as well as advanced genetic engineering [9,10,11,12]. However, since the ability of many genes to control multiple traits and a trait can be controlled by multiple genes, developing an N efficient crop without compromising other factors such as yield, biomass, and stress tolerance, is a challenging task. In that case, a complete understanding of gene regulation controlling complex traits such as NUE in genotype, tissue, and growth condition in a multi-trait dependent manner is required.

Many factors are known to be involved in the regulation of a particular gene expression. Alternative splicing (AS) is one such co-transcriptional mechanisms that fine-tunes the expression of many genes in plants under normal and stressful conditions [13,14,15]. It was estimated that almost 70% of intron-containing genes undergo AS in plants [16,17]. This percentage is expected to increase as the sequencing and bioinformatics analysis techniques are revolutionized, and more transcriptome data gathering in different tissues, conditions, and in a time-dependent manner in many plant species are performed. However, the transcriptome-wide AS analysis for exploring NUE in crop plants is still limited but starting to emerge. In the past transcriptome-wide AS under N stress is reported in rice [18] and maize [19].

Among various crops, rice is the third most produced and consumed food worldwide (http://statistics.amis-outlook.org/data/index.html#COMPARE; accessed on 25 December 2020). Moreover, being low NUE as compared to other cereals, it has become more significant to study the NUE trait in rice [20]. Improvement of NUE is possible to some extent by non-plant interventions such as the choice of fertilizer and the method/timing of its application and other crop management practices [21]. However, biotechnological interventions for increasing NUE are considered more important and it has been observed that plants show improved NUE in N limiting conditions or under N-starvation [22].

Several transcriptome-wide studies have been reported using high throughput sequencing technology in rice. However, these studies are largely limited to the identification of differentially expressed genes (DEGs) and their related pathways. To understand the deep insight of transcriptional dynamics of the NUE trait, in addition to gene expression, analysis at the AS level is also required. Therefore, in the current study, we: (i) performed reanalysis of previously generated transcriptome data [23] from root and shoot tissues of two rice genotypes IR-64 and Nagina-22 (N-22), under optimal N (N^+^) and N-starvation (N-); (ii) used high stringent criteria identified DEGs, differentially alternatively spliced (DAS) genes, differentially expressed transcripts (DETs), and differential transcript usage (DTU) transcripts among genotypes IR-64 and N-22, root and shoot tissues, and N^+^ and N- conditions were identified; and (iii) performed gene ontology (GO) functional enrichment analysis for DAS genes and DTU transcripts showing their involvement in several important metabolic pathways. Collectively, the outcome of the study will help our further understanding of transcriptional dynamics in response to the NUE trait in a genotype, tissue and condition dependent manner.

## 2. Materials and Methods

### 2.1. Prefetch and Preliminary Analysis of Transcriptome Data

Transcriptome data generated for two rice genotypes IR-64 and N-22 from the root and shoot tissues, under N^+^ and N^−^ conditions, was prefetched from NCBI database with accession numbers SRP131558 [23] and GSE147158 as two biological replicates. The prefetched reads were converted to fastq format using “—fastq-dump” and “–split-files” functions of the SRA-Toolkit [24]. The raw reads were quality checked and processed for adaptor trimming and low quality reads were removed by Trimmomatic v0.36 (http://www.usadellab.org/cms/?page=trimmomatic; accessed on 1 December 2020) [25]. Also, reads with less than a Phred33 quality score and with length less than 20 bp were removed. The quality of trimmed high-quality reads was further monitored using FastQC v0.11.7 (https://www.bioinformatics.babraham.ac.uk/projects/fastqc/; accessed on 1 December 2020). Further, high quality reads were quantified for transcript abundance by defining the “Transcript Per Million” (TPM) value for each transcript using Salmon v1.14.0 (https://combine-lab.github.io/salmon/; accessed on 1 December 2020) [26], using genome and transcriptome sequences downloaded from “The Rice Annotation Project Database” (RAP-DB, http://rapdb.dna.affrc.go.jp/; accessed on 1 December 2020) [27], as reference and indexing.

### 2.2. Bioinformatics Analysis for Differential Expression of Genes and Transcripts

A stringent 3D-RNA-Seq (https://3drnaseq.hutton.ac.uk/app_direct/3DRNAseq/; accessed on 12 December 2020) [28] analysis was performed to identify the DEGs, DAS genes, DETs and DTU transcripts in the transcriptome data. The “TPM” value generated by salmon quantification at transcript and gene level were passed to the 3D-RNA-Seq pipeline where initial gene length correction was performed by the “lengthScaledTPM” parameter of tximport (https://3drnaseq.hutton.ac.uk/app_direct/3DRNAseq/; accessed on 12 December 2020) [29]. Next, the transcripts with low quality were filtered out by selecting counts per million (CPM) value cut off 1 and number of samples 8 out of 16 (Table 1). The normalization of gene expression was performed using the trimmed mean of M-values (TMM) function of edgeR (https://3drnaseq.hutton.ac.uk/app_direct/3DRNAseq/; accessed on 12 December 2020) [30]. A principal component analysis (PCA) was performed before and after removing the batch effect among the biological replicates. Then normalized read counts were transferred to the limma (https://3drnaseq.hutton.ac.uk/app_direct/3DRNAseq/; accessed on 12 December 2020) [31], to identify the DEGs, DAS genes, DETs and DTU transcripts. In limma log2fold-change of CPM value ≥ 1 and Benjamini–Hochberg (BH) adjusted P-value ≤ 0.01 were assigned for DEGs and DETs. For DAS genes an additional criterion of the delta percentage spliced (ΔPS) ≥ 0.1 of an alternative splice isoform compared to the total gene expression within a contrast group was used.

### 2.3. Gene Ontology Functional Enrichment Analysis

Next, gene ontology (GO) functional enrichment analysis was performed using “The Gene Ontology Consortium” (http://geneontology.org/; accessed on 19 December 2020) [32,33], for DAS genes and DTU transcripts. All the significant DAS genes and DTU transcripts among all contrasting groups were passed through GO functional enrichment analysis for biological processes (BP), molecular functions (MF), and cellular components (CC). Additionally, DAS genes and DTU transcripts showed enrichment in which the protein class was also estimated. To get the significant functional enrichment for GO terms Fisher’s exact test was used and the false discovery rate was kept less than 5% (FDR ≤ 0.05).

### 2.4. Isoform Switch Analysis in 3D-RNA-Seq Pipeline

Isoform switches (ISs) occur when the relative abundance of a pair of transcripts reverse under different conditions [28]. The Iso-kTSP function of the 3D-RNA-Seq pipeline was used to identify the significant ISs under N^+^ and N^−^ conditions in both the genotypes and tissues. The raw ISs generated by average expression values of transcripts were filtered to significant ISs by using default criteria including probability cutoff = 0.5, difference cutoff = 1, *p*-value ≤ 0.01, minimum time in interval = 2, and correlation cutoff = 0 [34].

### 2.5. Alternative Splicing (AS) Transcriptional Landscape Visualization

The high-quality reads were also mapped to the reference genome using HISAT2 v2.2.1 (http://daehwankimlab.github.io/hisat2/; accessed on 19 December 2020) [35]. The alignment file generated in the BAM format for each sample library were passed to stringtiev2.1.4 [36], for reference based transcriptome assembly generated for each sample library. Further, the annotated transcripts were then passed to the AStalavista (http://astalavista.sammeth.net/; accessed on 26 December 2020) [37] to visualize the AS events landscape.

## 3. Results

### 3.1. Normalization and Principal Component Analysis (PCA) of Transcriptome Data

The TPM values assigned to genes and transcripts using Salmon [26] were uploaded to the 3D-RNA-Seq pipeline [28]. Among all, 16,600 genes and 18,175 transcripts were found to be expressed after filtering and mean variation analysis in the transcriptome data (Figure S1). Statistics of the number of reads, expressed genes, expressed transcripts, and parameters used in preliminary analysis is provided in Table 1. The transcriptome data was normalized using the weighted TMM value which is widely used in differential expression studies of transcriptomic data [28] (Figure S2).

The PCA after removing batch effects of the biological replicates suggested differential expression at gene and transcript levels occurred in tissues and condition dependent manner in rice (Figure S3). For instance, a total variance of 40.44% and 38.32% were detected at gene and transcript level, respectively, among the root and shoot tissues in both the varieties (IR-64 and N-22) (Figure S3). Moreover, N^−^ is another major factor behind the differential expression (DE) of many genes and transcripts suggested by the total variance of 16.7% and 15.98% at gene and transcript level, respectively (Figure S3). Overall, PCA analysis suggests that the DE of genes and transcripts occurs largely in a tissue dependent manner (root and shoot) in rice under N^−^ condition.

### 3.2. Genotype Dependent Differentially Expressed Genes/Transcripts, Alternative Spliced (DAS) Genes and Transcripts Usage (DTU) Transcripts

**Figure 1:**
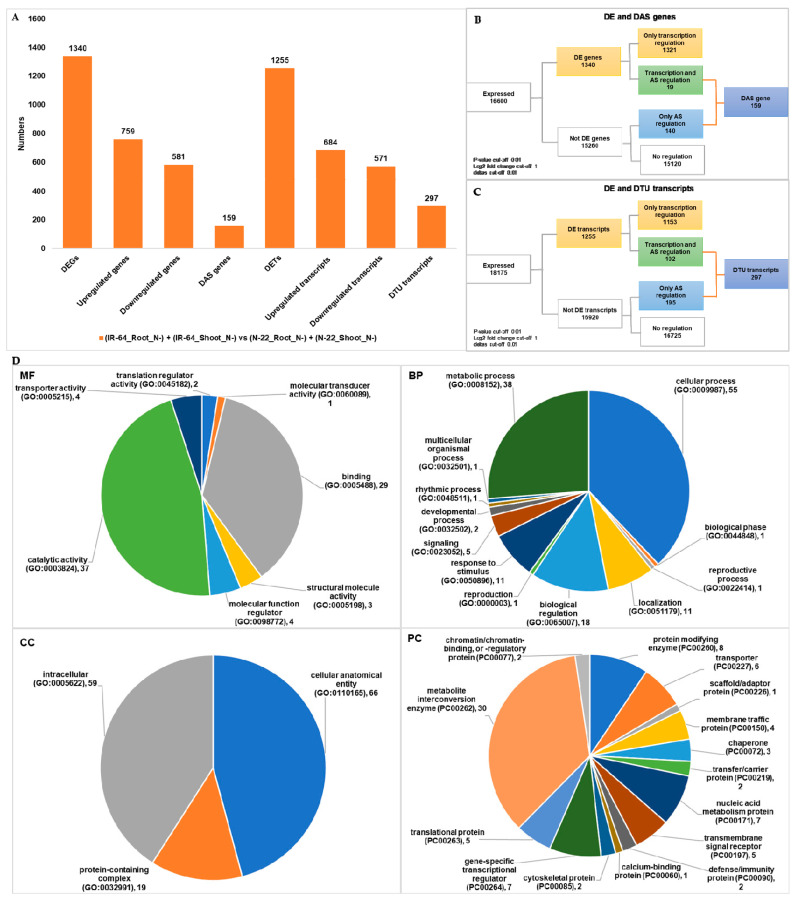
Genotype dependent differentially expressed genes (DEGs), differentially alternative spliced (DAS) genes, differentially expressed transcripts (DETs), and differentially transcript usage (DTU) transcripts. (**A**) Bar graph representing the statistics of genes and transcripts that undergo differential expression (DE) and alternative splicing (AS) in genotype dependent comparison (IR-64 versus N-22) under N-starvation (N^−^). (**B**) Union statistics between DEGs and DAS genes. (**C**) Union statistics between DETs and DTU transcripts. (**D**) Gene ontology (GO) term functional enrichment analysis in MF: molecular functions; BP: biological processes, CC: cellular components, and PC: protein class, for DAS genes and DTU transcripts in genotype dependent comparison (IR-64 versus N-22) under N-starvation (N^−^).

Transcriptome data of two indica rice genotypes IR-64 and N-22 were used in the current study. Since IR-64 is high yield N efficient and N-22 is upland tall cultivar [23,38], many genes showing DE and DAS under N- were anticipated [23,38]. Therefore, we employed 3D-RNA-Seq analysis [28] with high stringent criteria to identify DEGs, DETs, DAS genes, and DTU transcripts under N- conditions among these two genotypes of rice. Firstly, contrast group IR-64 (root and shoot) versus N-22 (root and shoot) under N- conditions were used for genotype dependent comparative analysis. Among 16,600 expressed genes, 1340 DEGs, and 159 DAS genes (Figure 1A; Table 2) and among 18,175 different transcripts 1255 differentially expressed (DETs), and 297 differential transcript usage (DTU) transcripts were identified (Figure 1A; Table 2). The list of all genotype dependent N- modulated DEGs, DAS genes, DETs and DTU transcripts is provided in a supplementary file (Table S1). In comparison to IR-64, among 1340 DEGs, 759 genes upregulated whereas 581 downregulated in N-22 genotype in response to N^−^ (Figure 1A). Similarly, at transcript level, out of 1255, 684 transcripts upregulated and 571 downregulated in N-22 as compared to IR-64 (Figure 1A). Surprisingly, the genes and transcripts which DE rarely underwent DAS and DTU, respectively, since a small portion of common genes and transcripts were observed between DEGs and DAS genes and DETs and DTU transcripts, respectively, (Figure 1B,C). This further suggests that under N- condition, DAS genes and DTU transcript expressions are independent to overall gene and transcript expression and are crucial not to be overlooked for important traits such as NUE. We further wanted to understand the role of genotype dependent DAS genes and DTU transcripts in rice under N- condition. Therefore, we performed gene ontology (GO) functional enrichment analysis using “The Gene Ontology Consortium” (http://geneontology.org/; accessed on 19 December 2020) [32,33], for DAS genes and DTU transcripts (Figure 1D). Interestingly, DAS genes and DTU transcripts are significantly enriched (FDR ≤ 0.05) in various metabolism processes including N and amino acid metabolisms (Figure 1D). As previously suggested, the DEGs under N- are found to be enriched in starch metabolism [23]; the involvement of DAS genes and DTU transcripts in additional macromolecule metabolic processes gives a new insight of AS and TU under N- condition in rice. Furthermore, RNA related catalytic activity and cellular anatomy (Figure 1D) related DAS genes and DTU transcripts are highly enriched, suggesting the role of RNA processing and different cellular components involved in N metabolism in rice. Overall, the comparative analysis between IR-64 and N-22 suggests both genotypes show different response to N- at the gene and transcription level.

### 3.3. Tissue Dependent Differentially Expressed Genes/Transcripts, Alternative Spliced (DAS) Genes and Transcripts Usage (DTU) Transcripts

**Figure 2:**
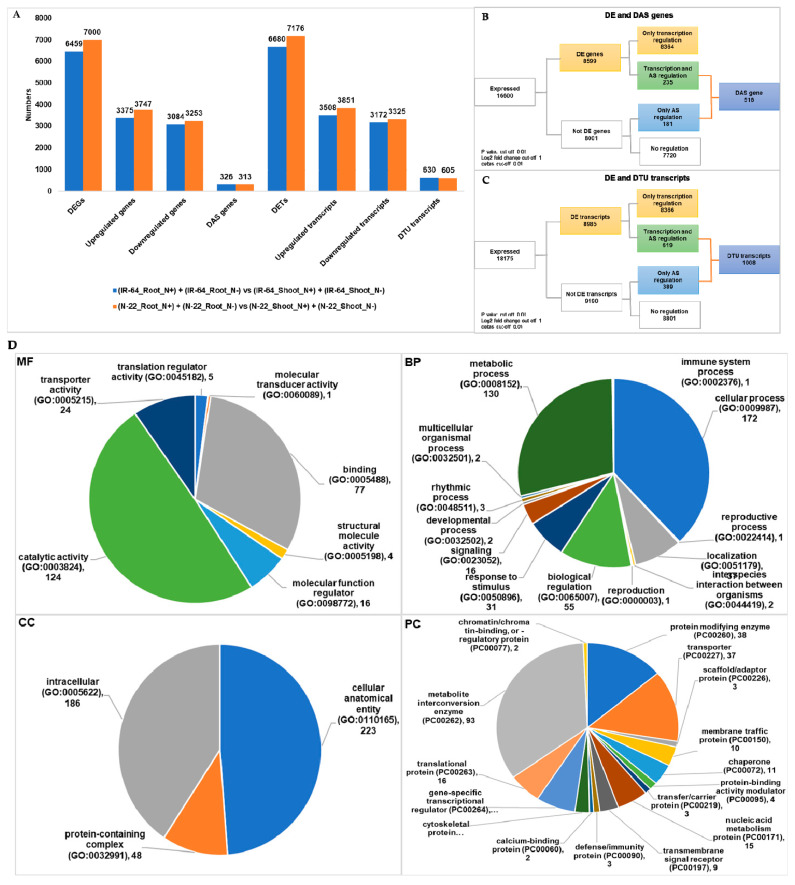
Tissue dependent differentially expressed genes (DEGs), differentially alternative spliced (DAS) genes, differentially expressed transcripts (DETs), and differentially transcript usage (DTU) transcripts. (**A**) Bar graph representing the statistics of genes and transcripts undergoing differential expression (DE) and alternative splicing (AS) in tissue dependent comparison (root versus shoot) under N-starvation (N^−^). (**B**) Union statistics between DEGs and DAS genes. (**C**) Union statistics between DETs and DTU transcripts. (**D**) Gene ontology (GO) term functional enrichment analysis in MF: molecular functions; BP: biological processes, CC: cellular components, and PC: protein class, for DAS genes and DTU transcripts in tissue dependent comparison (root versus shoot) under N-starvation (N^−^).

Since root and shoot tissues behave differently in response to N^−^ and involved different mechanism of metabolism [23], many genes and transcripts were expected to show DE in a tissue dependent manner. Therefore, next we identified tissue dependent DEGs, DAS genes, DETs and DTU transcripts by comparing root versus shoot transcriptome in both the genotypes IR-64 and N-22 under N- condition (Table S1 and Table 2). Among 16,600 expressed genes under N^−^, 3375 genes were found to be upregulated, and 3084 genes downregulated in IR-64 shoots as compared to IR-64 roots, whereas, 3747 genes were found to be upregulated, and 3253 genes downregulated in N-22 shoots as compared to N-22 roots (Figure 2A). Similarly, among 18,175 expressed transcripts 3508 upregulated, and 3172 downregulated in IR-64 shoots as compared to IR-64 shoots, whereas, 3851 transcripts upregulated, and 3325 transcripts downregulated in N-22 shoots as compared to N-22 roots (Figure 2A). Interestingly, changes at the transcriptional level in terms of gene and transcript expression between root and shoot tissues are very high (Figure 2A), as compared to the transcriptional level observed among genotypes (Figure 1A). Like DEGs and DETs, the number of DAS genes and DTU transcripts are also observed to be higher among tissues (Figure 2A) than genotypes (Figure 1A). Moreover, the number of DAS genes and DTU transcripts observed between root and shoot tissues were very high as compared to genotypes of rice. Among root and shoot tissues 516 genes underwent DAS and 1008 transcripts underwent DTU (Figure 2B,C). The overlap between DEGs and DAS genes, as well as DETs and DTU transcripts, were observed in tissue dependent comparison more than the genotype dependent comparison. It was observed that the overall overlap between genes and transcripts remained low, suggesting AS is an independent mechanism and modulated by N^−^ in a tissue dependent manner. Next, like in genotype comparison, the tissue comparison GO functional enrichment analysis was performed for DAS genes and DTU transcripts. Interestingly, many DAS genes and DTU transcripts were found to be enriched with BP that involved metabolism of organic compounds, phosphate containing compounds and nitrogen compounds (Figure 2C). Also, many genes found to be involved in biosynthesis such as organic compounds and other macromolecules biosynthesis (Figure 2C). Tissue dependent DAS genes and DTU transcripts were also found enriched in many MF such as various binding and catalytic activities (Figure 2C) and CC including cellular membrane (Figure 2C). The tissue level comparison (root versus shoot) in rice under N^−^, highlights the role of DAS in transcription regulation in a tissue dependent manner.

### 3.4. Condition Dependent Differentially Expressed Genes/Transcripts, Alternative Spliced (DAS) Genes and Transcripts Usage (DTU) Transcripts

**Figure 3:**
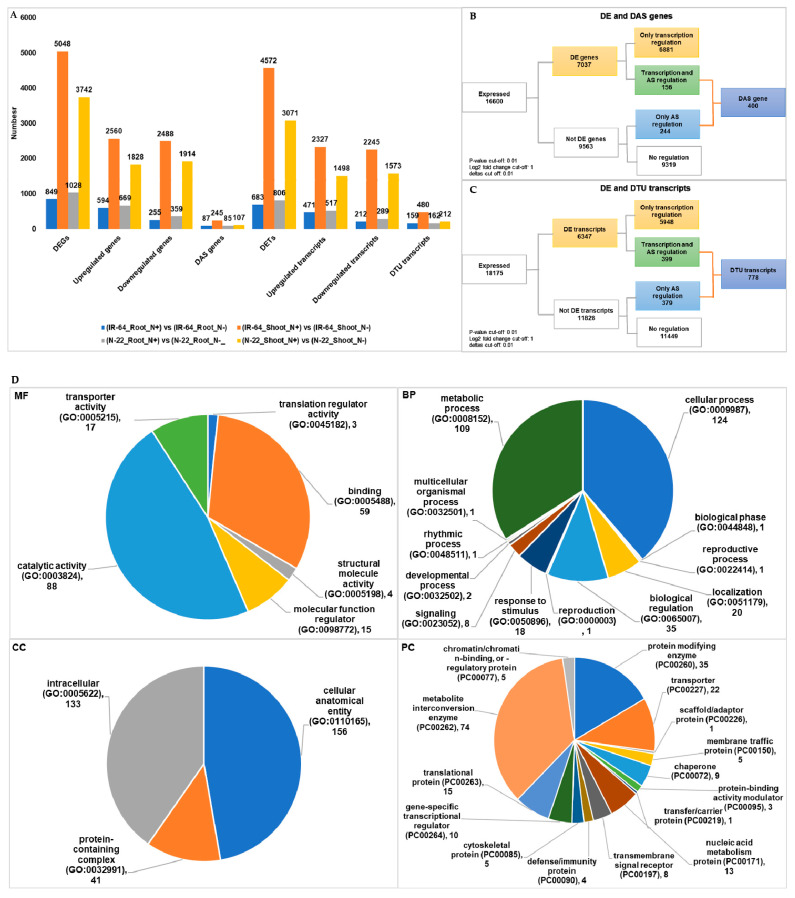
Condition dependent differentially expressed genes (DEGs), differentially alternative spliced (DAS) genes, differentially expressed transcripts (DETs), and differentially transcript usage (DTU) transcripts. (**A**) Bar graph representing the statistics of genes and transcripts that underwent differential expression (DE) and alternative splicing (AS) in condition dependent comparison (N^+^ versus N^−^) in different contrast groups. (**B**) Union statistics between DEGs and DAS genes. (**C**) Union statistics between DETs and DTU transcripts. (**D**) Gene ontology (GO) term functional enrichment analysis in MF: molecular functions; BP: biological processes, CC: cellular components, and PC: protein class, for DAS genes and DTU transcripts in condition dependent comparison (N^+^ versus N^−^) in different contrast groups.

Next, we wanted to understand how both the genotypes (IR-64 and N-22) and tissues (root and shoot) behave at gene and transcript levels under N^+^ and N^−^ conditions. Towards this goal we identified DEGs, DAS genes, DETs, and DTU transcripts in the individual contrast groups including (i) IR-64_Root_N^+^ vs. IR-64_Root_N^−^; (ii) IR-64_shoot_N^+^ vs. IR-64_shoot_N^−^; (iii) N-22_Root_N^+^ vs. N-22_Root_N^−^; and (iv) N-22_Shoot_N^+^ vs. N-22_Shoot_N^−^ (Table 2). Among all expressed genes, in total we identified 7037 DEGs and 400 DAS genes which change their expression under N- in comparison to N^+^ (Table S1 and Table 2; Figure 3A,B). Interestingly, the numbers of DEGs and DETs in shoots were identified more than roots, under N^−^ in both the genotypes (Table 2; Figure 3A). In IR-64 roots 594 genes upregulated and 255 downregulated under N- whereas in shoots 2560 genes upregulated and 2488 genes downregulated (Figure 3A). Likewise, in N-22 roots 669 genes underwent upregulation and 359 downregulation, whereas, in shoots 1828 genes underwent upregulation 1194 downregulation (Figure 3A). Similar pattern was observed in DETs as well. For instance, in IR-64 roots 471 transcripts were upregulated and 212 downregulated, whereas in shoots 2327 transcripts were upregulated and 2245 downregulated, respectively, under N^−^ (Figure 3A). In N-22 roots 517 transcripts were upregulated and 289 downregulated, whereas in shoots 1498 transcripts were upregulated and 1573 downregulated, respectively, under N^−^ (Figure 3A). Like previous results, in condition dependent comparison, only few (156 out of 400) DAS genes undergo DE (Figure 3B). At the transcripts level, among 778 DTU transcripts 399 showed DE in a condition dependent manner (Figure 3C). Since a large number of DEGs, DAS genes, DETs, and DTU transcripts were identified in a tissue dependent manner, we further examined the DAS genes and DTU transcripts for GO functional enrichment. The condition dependent DAS genes and DTU transcripts were significantly enriched in various important biological processes such as metabolic processes (cellular, nitrogen compounds, organic compounds, phosphorous containing compounds, protein, and macromolecular metabolism), biogenesis, gene expression, macromolecule modifications, and others (Figure 3D). Moreover, in molecular functions DAS genes and DTU transcripts are significantly enriched in various binding activities (organic cyclic compounds, ion, ATP, nuclease, and nucleic acid binding activities), catalytic activity, hydrolase activity, kinase activity, and others (Figure 3D). In cellular components cellular and intracellular anatomy, membrane bound organelles, and plasma membrane related genes were highly enriched (Figure 3D).

Next, we looked at the isoform switching (IS) in our datasets. IS is an important mechanism that occurs when a pair of transcript isoforms reverse in their abundance shifts from one condition to another [34]. ISs are crucial to be observed under N^−^ conditions since many transcript isoforms change their expression in a condition dependent manner. We also identified significant 99 ISs among different conditions (Table S2) in our analysis. Many isoform switches occurred between N^+^ and N^−^ conditions in the transcripts, which play an important role managing N^−^ conditions.

### 3.5. N-starvation (N^−^) Modulates Different Alternative Splicing (AS) Events.

**Figure 4:**
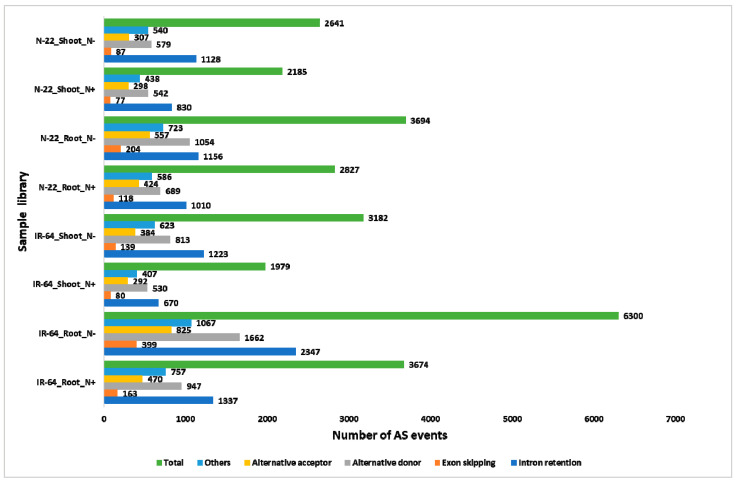
Alternative splicing (AS) events predicted in different contrast groups. *x*-axis: the number of AS events predicted in *y*-axis: sample library. The main AS events are intron retention (IR); exon skipping (SE); alternative donor (alt 5’ splice site); alternative acceptor (alt 3’ splice site).

In plants under stressful conditions different AS events change their ratio to fine tune the expression of many genes and transcript abundance [14,39]. Therefore, we were also interested in seeing the AS transcriptional landscape in terms of events visualized under N^−^ conditions. Interestingly, the number of AS events increases, under N^−^ conditions, as compared to N^+^ in all the sample libraries (Figure 4). In AS event analysis intron retention (IR) was observed as the major contributor of AS in both the genotypes (IR-64 and N-22) and in both tissues (root and shoot) under N^+^ and N^−^ conditions (Figure 4). These findings are in line with previous work in plants where IR is the most common AS event [16,17,39]. Next to IR, alternative donor (alt 5’ splice site), followed by alternative acceptor (alt 3’ splice site) were observed to be the main contributors towards AS in rice under N^+^ and N^−^ conditions (Figure 4). Like other plants species, exon skipping (SE) is the least represented AS event in rice. Collectively, the results from AS event analysis further supports the notion that under N^−^, AS is crucial in rice and should not to be overlooked in studies involving NUE traits.

## 4. Discussion

The extensive use on N fertilizers to increase crop yield creates a potential threat to the environment and economy of the agriculture sector [40,41]. To minimize the use of N fertilizers, crop varieties with high NUE need to be developed that further require a complete understanding of molecular mechanism of NUE traits. Among all major crops, rice, being less N efficient and major staple food worldwide [20], attracts the attention of research community worldwide to study NUE traits. Many attempts have been made previously to identify the N responsive genes in rice using transcriptome sequencing and microarray-based studies [18,23,42,43]. However, the data generated in these studies are restricted to the finding of DEGs and related pathways. Since, under stressful conditions, plants undergo extensive AS to fine tune the expression of various genes and the abundance of transcripts [15]. Therefore, it is essential to understand transcriptional dynamics at the AS level under different stress conditions in plants. Also, it was previously shown that the absence and presence of mineral nutrients (Fe, Mn, Cu, P, and Zn) changes the pattern of AS to regulate the expression of many genes in rice [44]. Therefore, in the current study we utilized the recently developed 3D-RNA-Seq pipeline [28] to explore the transcriptome data from the root and shoot tissues of two rice varieties (IR-64 and N-22) under N^+^ and N^−^ conditions [23,38], for AS analysis.

Being genetically different, IR-64 and N-22, respond differently under N^−^ condition [23]. Since IR-64 has high NUE as compared to N-22, under N^−^ the later showed large number of genes that underwent upregulation (Figure 1). In addition to DEGs and DETs we also identified 159 DAS genes, and 297 DTU transcripts among both the genotypes (Figure 1; Table 2). This suggests that both rice genotypes are not only genetically different but there is various transcriptional changes occurs under N^−^ conditions, among them. Similar observation was made in comparative analysis between different tissues (root versus shoot; Figure 3; Table 2) and under different conditions of nitrogen (N^+^ versus N^−^; Figure 3; Table 2). In line of previous finding in rice [23], we also observed an increased number of DEGs and DETs on comparing root to shoot tissues under N^−^ condition (Figure 2; Table 2) in both the genotypes. In general, the transcriptional dynamics changes among tissues under N^−^ conditions are higher than the changes among the genotypes. We identified a greater number of DAS genes (516) and DTU transcripts (1008) in dependent tissue than genotype under dependent comparative analysis in response to N- conditions in rice. Moreover, under different conditions (N^+^ versus N^−^) in individual genotype and tissue, these numbers increase significantly. Like previously reported [23] we also observed that the number of DEGs and DETs are increased in shoot tissue compared to root tissue in both the genotypes (Figure 3; Table 2). The DAS genes (400) and DTU transcripts (778) numbers were also higher in root and shoot tissues when compared between N^+^ and N^−^ conditions (Figure 3; Table 2). It is fascinating that most transcriptional dynamics changes are observed under N^−^ conditions in genotype, tissue, and condition dependent manner. However, the observed overlap between DEGs and DAS genes, and DETs and DTU transcripts is very small in all contrasting groups (*p*-value ≤ 0.01). This further supports the previous findings in rice [44], where the author found a very small overlap among DEGs and AS genes in roots under absence and presence of mineral nutrients including Fe, Mn, P, Cu, and Zn. Here in the current study, we expand our understanding further to DAS genes and DTU transcripts in rice root and shoot tissues. Collectively, our results on comparative analysis suggests that AS and TU are very crucial to an in depth understanding of the molecular mechanism of NUE traits in rice and other unexplored crop plants.

The GO terms suggest that in DAS genes and DTU transcripts identified in response to N^−^ in rice are significantly enriched in many important biological processes, molecular functions, and cellular components. In the previous study DEGs were found to be involved in starch metabolism and chloroplast under N^−^ in rice [23]. In the current study, we observed that the majority of DAS genes and DTU transcripts are significantly enriched in various metabolic processes, including starch, proteins and other N containing compounds metabolisms. Under limiting supply of nutrients (N in our case), the plants needs to accelerate their metabolic activities, which requires upregulation or downregulation of metabolism related genes [44]. We further suggest from our analysis that AS adds another layer of fine tuning to the regulation of genes related to metabolism in rice under N^−^. Additionally, other GO terms which were found to be dominant among DAS genes and DTU transcripts include catalytic and binding activities, and cellular anatomy. We also looked at the protein class to understand which proteins and/or enzymes are enriched in DAS genes and DTU transcripts (Figure 1, Figure 2 and Figure 3). The metabolite interconversion enzymes such as hydrolase, isomerase, ligase, lyase, oxidoreductase, and transferase followed by protein modifying enzymes were found significantly enriched in protein class categories. N^−^ stress transport of N from one cell to another is modulated by several transporter proteins present in the cell membrane. The transporter, transfer/carrier, and membrane traffic proteins were also found to be dominating in the DAS genes and DTU transcripts. Additionally, stress related chaperons and defense/immunity proteins were also enriched in DAS genes and DTU transcripts. Overall, GO terms assigned to DAS gens and DTU transcripts support the notion that various metabolism related genes undergo AS and TU in rice under N^−^.

Collectively, pleiotropic traits such as NUE are influenced by several other traits, including availability of macro- and micro-nutrients, plant–soil interaction, and, environmental factors [45,46]. The dynamic and extensive changes in the transcriptome are also likely to affect other abiotic and biotic factors. Water and N are considered as the main factors that are known to interact with each other and N metabolism affects many significant physiological and biochemical changes that occur because of drought stress, such as opening and closing of stomata [47]. Also crop yield is linked with N and water availability to plant, as it is observed that photosynthesis decreases more effectively than transpiration with reduced N availability [48]. The availability and accumulation of other minerals in soil along with plant root–soil interactions may also affect the NUE in different crops as biochemical pathways are known to be interrelated with each other. Moreover, interspecific interactions and soil nitrogen supply levels affect intercropping productivity in various important crops [49]. However, the effect of interspecific interaction on nutrients availability and yield has not been explored much in rice.

We also observed the difference in AS events abundance among different contrasting groups. Since IR is the most frequent AS event in plants [14,39], we also observed the same pattern in rice under both N^+^ and N^−^ conditions in all samples (Figure 4). Under N^−^ in each individual sample there is an increase in number of AS events as well as IR followed by an alternative donor (alternative 5’ splice sites), which is in line with previous AS studies in plants under normal as well as stress conditions [14,16,17,39].

By comparing the expression profiles at gene and transcripts levels in two contrasting genotypes IR-64 and N-22, and root and shoot tissues, for a NUE trait under N^+^ and N- conditions, we predicted genes and transcript with DE, DAS and DTU respectively. Based on the methodology, current study could be utilized as a reference for a large amount of transcriptome data generated and available in the public domain for various conditions in different plant species restricted to the identification of DEGs only. However, the simultaneous link of the pleotropic nature of the NUE trait with other factors is the major limitation of the current study.

## 5. Conclusions

The current study provides a comprehensive as well stringent analysis for genes and transcripts that underwent AS and TU, under N^−^ in rice. The genotype, tissue, and condition dependent DAS genes and DTU transcripts identified in the study will further help the research community in understanding the transcriptional dynamics of NUE traits in rice and other important crops, although achieving high NUE in any crop is a challenging and a desirable trait both from an economical and environmental viewpoint. Gaining insights into the genetic basis of NUE paves the way for assessment of this trait in the near future. However, due to the complexity of the NUE trait at the cell, tissue, and plant levels, the emerging research may take advantage of proteome and metabolome data analysis using advanced computational and bioinformatics tools along with other transcriptomics studies. Although different omics approaches have emerged in the past decade as an essential tool to closely understand plants biological systems. Therefore, by analyzing altered expression of various genes involved in N uptake, assimilation and remobilization along with studying the convergences of proteins and other metabolites during N starvation may provide more insights. Additionally, machine learning for high-throughput stress phenotyping tools will further advance our understandings of NUE trait in other cultivars along with rice [50].

## Figures and Tables

**Table 1:** Statistics of preliminary transcriptome data analysis in the 3D-RNA-Seq pipeline.

Description	Number
Raw transcripts	33,195
Raw genes	28,120
Samples	16
Samples after merging seq-reps	8
Condition of interest	8
CPM cut-off	1
Min samples to CPM cut-off	8
Expressed transcripts	18,175
Expressed genes	16,600

**Table 2:** Statistics of significant differentially expressed genes (DEGs)/transcripts (DETs), alternative spliced (DAS) genes, and transcript usage (DTU) transcripts.

Contrast Group	DEGs	DAS Genes	DETs	DTU Transcripts
(IR-64_Root_N^−^) + (IR-64_Shoot_N^−^) vs. (N-22_Root_N^−^) + (N-22_Shoot_N^−^)	1340	159	1255	297
(IR-64_Root_N^+^) + (IR-64_Root_N^−^) vs. (IR-64_Shoot_N^+^) + (IR-64_Shoot_N^−^)	6459	326	6680	630
(N-22_Root_N^+^) + (N-22_Root_N^−^) vs. (N-22_Shoot_N^+^) + (N-22_Shoot_N^−^)	7000	313	7176	605
(IR-64_Root_N^+^) vs. (IR-64_Root_N^−^)	849	87	683	159
(IR-64_Shoot_N^+^) vs. (IR-64_Shoot_N^−^)	5048	245	4572	480
(N-22_Root_N^+^) vs. (N-22_Root_N^−^)	1028	85	806	162
(N-22_Shoot_N^+^) vs. (N-22_Shoot_N^−^)	3742	107	3071	212

## Data Availability

The data used in the current study is publicly available on NCBI under the accession numbers SRP131558 and GSE147158.

## Supplementary Materials

The following are available online at https://www.mdpi.com/article/10.3390/life11040285/s1, Figure S1: The mean variance trends before (raw counts) and after removing (filtered counts) low expressed genes (a) and transcripts (b). Filtering criteria include read counts per million (CPM) = 1 and sample size = 8, Figure S2: Distribution of read counts before and after normalization. The groups X1 and X2 represent the biological replicates. Control = N^+^ and Treatment = N^−^. IR64 = IR-64, and N.22 = Nagina-22 (N-22), Figure S3: The principal component analysis (PCA) plot after removing batch effects from biological replicates at the gene and transcript levels in different contrasting groups. The groups X1 and X2 represent the biological replicates. Control = N^+^ and Treatment = N^−^; IR64 = IR-64, and N.22 = Nagina-22 (N-22), Table S1: List of differentially expressed genes (DEGs), differentially alternative spliced (DAS) genes, differentially expressed transcripts (DETs), and differentially transcript usage (DTU) transcripts, in genotype, tissues and condition dependent comparative analysis, Table S2: Significant isoform switches among different samples, Table S3: Statistics of significant differentially expressed genes (DEGs), and differentially alternative spliced (DAS) genes, identified in genotype, tissues and condition dependent comparative analysis.
