# Supplementary material for: Rice Transcriptome Analysis Reveals Nitrogen Starvation Modulates Differential Alternative Splicing and Transcript Usage in Various Metabolism-Related Genes
Source: Life (Basel). 2021 Mar 27;11(4):285. doi: 10.3390/life11040285 (PMC8066416; doi:10.3390/life11040285)
Supplement: Supplementary file 1 [file life-11-00285-s001.zip › Supplementary Figures.doc]

**Figure S1:** The mean variance trends before (Raw counts) and after removing (Filtered counts) low expressed genes (a) and transcripts (b). Filtering criteria includes reads counts per million (cpm) = 1 and sample size = 8.

**Figure S2:** Distribution of read counts before and after normalization. The groups X1 and X2 represents the biological replicates. Control = N+ and Treatment=N-. IR64 = IR-64, and N.22 = Nagina-22 (N-22).

**Figure S3:** The principal component analysis (PCA) plot after removing batch effects from biological replicates at gene and transcript levels in different contrasting groups. The groups X1 and X2 represents the biological replicates. Control = N+ and Treatment=N-; IR64 = IR-64, and N.22 = Nagina-22 (N-22).
